# Lamotrigine Augmentation of Serotonin Reuptake Inhibitors in Severe and Long-Term Treatment-Resistant Obsessive-Compulsive Disorder

**DOI:** 10.1155/2013/612459

**Published:** 2013-07-14

**Authors:** Manuel Arrojo-Romero, María Tajes Alonso, Jose de Leon

**Affiliations:** ^1^Department of Psychiatry, Complejo Hospitalario Universitario, 15701 Santiago de Compostela, Spain; ^2^Servicio de Salud Mental y Asistencia a Drogodependencia Servicio Gallego de Salud, 15703 Santiago de Compostela, Spain; ^3^Mental Health Research Center, Eastern State Hospital, Lexington, KY 40508, USA

## Abstract

The treatment recommendations in obsessive-compulsive disorder (OCD) after lack of response to selective serotonin reuptake inhibitors (SSRIs) include augmentation with other drugs, particularly clomipramine, a more potent serotonin reuptake inhibitor (SRI), or antipsychotics. We present two cases of response to lamotrigine augmentation in treatment-refractory OCD; each received multiple SRI trials over a >10-year period. The first patient had eleven years of treatment with multiple combinations including clomipramine and SSRIs. She had a >50% decrease of Y-BOCS (from 29 to 14) by augmenting paroxetine (60 mg/day) with lamotrigine (100 mg/day). The second patient had 22 years of treatment with multiple combinations, including combinations of SSRIs with clomipramine and risperidone. She had an almost 50% decrease of Y-BOCS (from 30 to 16) and disappearance of tics by augmenting clomipramine (225 mg/d) with lamotrigine (200 mg/day). These two patients were characterized by lack of response to multiple treatments, making a placebo response to lamotrigine augmentation unlikely. Prospective randomized trials in treatment-resistant OCD patients who do not respond to combinations of SSRIs with clomipramine and/or antipsychotics are needed, including augmentation with lamotrigine. Until these trials are available, our cases suggest that clinicians may consider lamotrigine augmentation in such treatment-resistant OCD patients.

## 1. Introduction

 There is general agreement in the obsessive-compulsive disorder (OCD) literature that the first line for pharmacological treatment is the selective serotonin reuptake inhibitors (SSRIs). This is supported by practical guidelines [1], meta-analysis [2], and treatment algorithms [3]. The recommendations after lack of response to SSRIs are not so well established but usually include augmentation with other drugs: (1) particularly clomipramine, a more potent serotonin reuptake inhibitor (SRI), or (2) antipsychotics [1–6]. More recently, augmentation with glutamatergic agents has also been recommended [3].

 Lamotrigine is an antiepileptic drug and mood stabilizer that has antiglutamatergic properties [7] and has been occasionally used in OCD treatment. Two small open studies used lamotrigine for treating obsessive symptoms, one in schizophrenia [8] and another in bipolar disorder [9]. A small open study [10] of augmentation of SRIs with 100 mg/day of lamotrigine in 8 patients reported negative results. A case report [11] of lamotrigine augmentation using up to 150 mg/day in a patient with a stable dose of clomipramine (225 mg/day) described a remarkable improvement. Bruno et al. [12] conducted a 16-week double-blind, randomized, and placebo-controlled trial of lamotrigine augmentation (up to 100 mg/day) in patients receiving SSRIs. The patient had an adequate SSRI trial for at least 12 weeks and was still having enough OCD symptoms, as determined by a Yale-Brown Obsessive-Compulsive Scale [13, 14] (Y-BOCS) score >16. This scale has a possible range of 0 to 40. At the end of the study, 85% (17/20) of the lamotrigine patients met response criteria of 25% improvement or greater in Y-BOCS total score compared with baseline versus none in the 20 placebo patients. 

 Here, we describe two cases of marked response to lamotrigine augmentation in open treatment, assessed by the same psychiatrist (the first author). These two cases are important because they include patients who were treatment-refractory and received multiple SRIs trials over a >10-year period, making the possibility of placebo response unlikely. 

## 2. Case Presentations

### 2.1. Case 1

This was a 24-year-old Spanish woman with family history of bipolar disorder on her maternal side. She retrospectively reported that her first symptoms started at the age of 8 when she had an obsessive fear that something would happen to her mother. At 12 years of age she was diagnosed with OCD; at that time she had developed religious obsessions and compulsions to kiss saints' pictures to avoid something wrong happening. During these 11 years, medications used included clomipramine (up to 225 mg/day), fluoxetine (up to 60 mg/day), fluvoxamine (up to 200 mg/day), paroxetine (up to 60 mg/day), lithium (up to 400 mg/day), bupropion (up to 300 mg/day), and unknown doses of buspirone, clonazepam, and lorazepam. She visited different psychiatrists in different areas of Spain, including an outpatient clinic specialized in OCD treatment.

 At the first visit she had a Y-BOCS score of 29 in spite of cognitive behavioral therapy (CBT) and pharmacological treatment with paroxetine (40 mg/day), lithium (400 mg/day), and clonazepam (1 mg at night). The predominant symptoms were obsessions of an aggressive, religious, and sexual nature. The psychiatrist proposed lithium discontinuation, an increase of paroxetine to 60 mg/day, and the addition of lamotrigine 25 mg/day in the first week with an increase of 25 mg/week to reach a dose of 100 mg/day in 4 weeks. After 5 weeks, at the second visit the patient was taking paroxetine (60 mg/day), lamotrigine (100 mg/day for 1 week), and clonazepam (1 mg at night). She had a remarkable improvement with a reduction >50% and a total Y-BOCS score of 14. Twelve weeks after the initial visit, at the third visit with the same CBT and pharmacological treatment, the patient reported a worsening of OCD symptoms (Y-BOCS total score = 19) due to psychosocial stressors (family problems and university exams). Due to her anxiety she had started to pinch her skin when her guilt feelings were worse. The psychiatrist added 50 mg/day of quetiapine-extended release. Sixteen weeks after the initial visit, at the fourth visit with the same CBT and taking paroxetine (60 mg/day), lamotrigine (100 mg/day), clonazepam (1 mg at night), and quetiapine-extended release (50 mg/day), the patient reported an improvement, the disappearance of anxiety associated with a decrease in psychosocial stress. The psychiatrist discontinued the quetiapine. Twenty-four weeks after the initial visit, at the fifth visit with the same CBT and taking paroxetine (60 mg/day), lamotrigine (100 mg/day), and clonazepam, the patient reported a complete disappearance of psychosocial stressors and improvement in OCD symptoms (Y-BOCS total score = 14). The patient reported that lamotrigine had helped more than any other treatment, and she had never felt as well since she started treatment at the age of 12. After another year of follow-up (1 year and 7 months after the initial visit), the patient continued to report mild OCD chronic symptoms with limited functional impairment to the point that the patient has been able to work for three months in another country, proving to herself that she can be an autonomous person in spite of her illness. As the patient is very insightful about her illness and treatment response, the psychiatrist asked her, on a Likert scale of 0–10, to rate lamotrigine's contribution to her improvement, and she responded 7 to 8. 

### 2.2. Case 2

This was a 46-year-old Spanish woman. She had no medical problems and no family history of psychiatric disorder. She had been diagnosed 22 years earlier and had received multiple treatments including fluoxetine (up to 60 mg/day), paroxetine (up to 40 mg/day), and bromazepam. At the age of 43 she was taking clomipramine (225 mg/day) and fluvoxamine (200 mg/day); risperidone (0.5 mg/day) was added. The patient took this combination for at least 6 months, and then she decided to discontinue risperidone due to its lack of efficacy. Over the last two years she had received the combination of clomipramine (225 mg/day), fluvoxamine (200 mg/day), and alprazolam (2 mg/day). She definitively was a treatment-resistant case and had no response to risperidone augmentation. As a matter of fact, with the then-current treatment including relatively high doses of both clomipramine and fluvoxamine, she had a Y-BOCS total score of 30 (possible range 0–40) [12, 13]. At that time, she had intrusive and repetitive thoughts about negative events, fear of hurting someone, and apprehension that something terrible might occur. Her compulsions included frequent checking rituals of the domestic gas valves or the house locks. She also had motor tics (her shoulders moving up). 

 The patient agreed to a lamotrigine augmentation trial after fluvoxamine discontinuation. Two weeks after fluvoxamine was discontinued, lamotrigine was added to clomipramine 225 mg/day and alprazolam 2 mg/day. The initial lamotrigine dose was 25 mg/day; it was increased 25 mg weekly until reaching 100 mg/day. Five weeks after the first lamotrigine dose and one week after reaching 100 mg/day of lamotrigine, she reported an improvement in compulsions, obsessions, and tics. The patient was delighted with the response and agreed to increase lamotrigine to 200 mg/day. By the time of the third visit, after 11 weeks of the lamotrigine trial and 5 weeks on the 200 mg/day dose, the tics had completely disappeared and the Y-BOCS score was 21. At the time of the last evaluation, after five months on lamotrigine and 3.5 months on the 200 mg/day dose, the tics continued to be absent and the Y-BOCS score had decreased to 16. No secondary effects were reported. It is important to stress that (1) the patient had 22 years of prior treatments including no obvious response to combinations of SSRI plus clomipramine and SSRI plus risperidone, making a placebo response to lamotrigine augmentation unlikely, and (2) the response to lamotrigine augmentation was dramatic with a 47% decrease in Y-BOCS total score and disappearance of the tics. 

## 3. Discussion 

 Two recent literature reviews [15, 16] of glutamatergic mechanisms in OCD describe why glutamatergic agents such as lamotrigine may help OCD. The list of glutamatergic drugs tried in OCD includes riluzole [15, 16], memantine [15], ketamine [15], glycine [15], sarcosine [15], N-acetylcysteine [15, 16], D-cycloserine [15, 16], topiramate [15], and lamotrigine [15, 16]. 

 Wu et al. [16] summarized the prior literature on the pivotal role of glutamate in corticostriatal-thalamocortical (CSTC) models of OCD. One of the leading OCD models is based on the balance between direct and indirect pathways within CSTC circuits. According to this theory, reciprocal interaction between direct (ultimately leads to thalamic stimulation of the cortex) and indirect (ultimately leads to thalamic inhibition of the cortex) pathways normally resulted in a dynamic balance with no one pathway predominating. Hyperactivity of the direct pathway or hypoactivity of the indirect pathway would disinhibit CSTC circuits and promote consequent release of hardwired behaviors (compulsions) and cognitions (obsessions) that were normally held in check. 

 The exact mechanism explaining why lamotrigine or other glutamatergic agents may be good augmentation strategies in OCD is not as important as the need for more prospective well-controlled trials demonstrating whether lamotrigine may help in all treatment-resistant OCD or at least in subgroups with glutamatergic abnormalities. OCD may be a heterogeneous disorder, and not all patients may have glutamatergic abnormalities [15]. In the first randomized, placebo-controlled trial of lamotrigine augmentation, Bruno et al. [12] studied patients for 16 weeks who were taking up to 100 mg/day of lamotrigine; their patients had lower baseline Y-BOCS scores (mean 26) than our two patients. It is important to stress that none of their placebo patients responded to placebo because moderate-to-severe OCD cases are not likely to respond to placebo. Our two patients were characterized by severe long-term OCD with lack of response to multiple treatments, making a placebo response to lamotrigine augmentation unlikely. 

 The first of our patients had multiple years of treatment with multiple drug combinations including SSRIs and clomipramine. She had more than 50% decrease in Y-BOCS score by augmenting paroxetine with lamotrigine up to 100 mg/day. The second patient had multiple years of treatment with multiple combinations including the combination of SSRIs and clomipramine and SSRIs and risperidone. After augmenting clomipramine with lamotrigine up to 200 mg/day, she had almost a 50% decrease in Y-BOCS baseline score and disappearance of tics.

 Prospective randomized trials in patients such as those presented in this paper with treatment-resistant OCD who do not respond to combinations of SSRIs with clomipramine and/or antipsychotics are needed. Lamotrigine may be an ideal candidate for these trials.
